# Associations of alcohol with the human gut microbiome and prospective health outcomes in the FINRISK 2002 cohort

**DOI:** 10.1007/s00394-025-03668-z

**Published:** 2025-04-11

**Authors:** Kari Koponen, Daniel McDonald, Pekka Jousilahti, Guillaume Meric, Michael Inouye, Leo Lahti, Teemu Niiranen, Satu Männistö, Aki Havulinna, Rob Knight, Veikko Salomaa

**Affiliations:** 1 Department of Public Health and Welfare, Finnish Institute for Health and Welfare (THL), P.O. Box 30, Helsinki, 00271 Finland; 2 Department of Bacteriology and Immunology, University of Helsinki, Helsinki, Finland; 3 Department of Pediatrics, University of California San Diego, La Jolla, San Diego, CA USA; 4 Cambridge Baker Systems Genomics Initiative, Baker Heart and Diabetes Institute, Melbourne, Australia; 5 Department of Infectious Diseases, Central Clinical School, Monash University, Melbourne, Australia; 6 Department of Cardiovascular Research, Translation, and Implementation, La Trobe University, Melbourne, VIC Australia; 7 Department of Cardiometabolic Health, University of Melbourne, Melbourne, VIC Australia; 8 Cambridge Baker Systems Genomics Initiative, Department of Public Health and Primary Care, University of Cambridge, Cambridge, UK; 9 Department of Computing, University of Turku, Turku, Finland; 10 Department of Medicine, Turku University Hospital and University of Turku, Turku, Finland; 11 Institute for Molecular Medicine Finland, FiMM-HiLIFE, Helsinki, Finland; 12 Center for Microbiome Innovation, University of California San Diego, La Jolla, San Diego, CA USA; 13 Department of Computer Science & Engineering, University of California San Diego, La Jolla, San Diego, CA USA; 14 Shu Chien - Gene Lay Department of Bioengineering, University of California San Diego, La Jolla, San Diego, CA USA; 15 Halıcıoğlu Data Science Institute, University of California San Diego, La Jolla, San Diego, CA USA

**Keywords:** Alcohol, Gut microbiome, Epidemiology, Prospective

## Abstract

**Background and aims:**

Alcohol remains a global risk factor for non-communicable diseases with the gut microbiome emerging as a novel elucidator. We investigated how gut microbiome associates with alcohol on population level, if there is mediation reflected in health outcomes, and how functional potential is related.

**Methods:**

Our sample consisted of 4575 shallow-shotgun sequenced fecal samples from the FINRISK 2002 cohort (25-74yrs., 52.5% women). Alcohol (g 100% alcohol/week) use was self-reported. Diversity and differential species abundances were analyzed using multiple linear regression. Compositional differences were analyzed using PERMANOVA, and prospective associations with Cox-regression. Connections between alcohol, microbiome, inflammatory markers, and outcomes were assessed using serial mediation. Functional associations were assessed using KEGG-orthologies and multiple linear regression.

**Results:**

High-risk alcohol consumers had significantly lower bacterial diversity when compared to low-risk consumers (mean±SD:4.04±0.41 vs. 4.11±0.43, *p* = 9.56 × 10^− 4^). Alcohol also associated with significant shifts in overall composition (PERMANOVA; *p* ≤ 1.00 × 10^− 4^) and differential abundances of 344 species (ANCOM-BC2; q ≤ 0.05). These shifts were characterized by an increase in relative abundances of Gram-negative bacteria, the top genera of which were *Bacteroides* and *Prevotella*, and a decrease in putatively beneficial species in genera such as *Lactobacillus*, *Bifidobacterium*, and* Akkermansia*. Prospective associations with all-cause mortality (HR:1.12 [1.02—1.23]), and liver disease (HR:1.53 [1.22—1.92]) were observed. The association between alcohol and liver disease had a mediating link via a proinflammatory beta-diversity principal coordinate (OR:1.04 [1.001—1.10]). Functional associations were observed with 1643 KO-groups (q < 0.05, n_positive_=431, n_negative_=1212). Antioxidative and gut integrity maintaining functions were diminished and lipopolysaccharide synthesis enriched.

**Conclusions:**

Alcohol use is associated with community-level shifts in composition towards enriched Gram-negative bacteria, and diminished levels of putatively beneficial bacteria. Alcohol use associates with a proinflammatory gut microbiome profile that mediates alcohol’s effect on incident liver disease risk, possibly via increased proliferation of endotoxins through the gut epithelial lining.

**Supplementary Information:**

The online version contains supplementary material available at 10.1007/s00394-025-03668-z.

## Introduction

Alcohol is a widely consumed beverage worldwide with well-known health hazards [1]. High alcohol use is among the top 10 global risk factors by share of attributable disability-adjusted life years and is one of the leading risk factors for a wide variety of non-communicable diseases [2]. For instance, high alcohol use has been linked with increased risks for all-cause mortality, cardiovascular diseases (CVD, excl. myocardial infarction), type-2 diabetes (T2D), and liver diseases (LD), although moderate use can be a protective factor for some conditions [3–5]. However, there is some heterogeneity in these outcomes, and all the underlying mechanisms are not yet fully understood. The gut microbiome is one emerging factor under study for explaining this variability [6–11].

The term gut microbiome entails all the organisms, i.e., bacteria, archaea, viruses, fungi, and protozoa inhabiting the epithelial lining and lumen of the intestine along with all their various genetic capabilities. The intestinal ecosystem is highly dynamic and constantly subject to change in its prevailing environment, which in turn is reflected on the composition and functions of its inhabitants [12, 13]. Ingested food, beverages, medications, stress-levels, exposure to exogenous microbes, can all impact the make-up and function of the gut microbiome. It is therefore no surprise that the gut microbiome is linked to a multitude of health outcomes as well, such as CVD [14, 15], T2D [16, 17], and LD [18].

Improvements in high-throughput sequencing and statistical methodology, followed by the emergence of large-scale microbiome datasets, have facilitated epidemiological assessment between the gut microbiome and human health. Existing literature on the associations of alcohol with the gut microbiome, however, remains limited in terms of study size, representativeness, sequencing technology, and lack of prospective follow-up. In this study, with access to microbiome data from the large and well-characterized FINRISK 2002 cohort, we set out to examine epidemiologic associations between the human gut microbiome, usage of alcohol, and health outcomes frequently linked with its use.

Our aims were to see (i) how the use of alcohol is associated with gut microbiome diversity and composition on the general population level. (ii) Whether observed associations with health outcomes (CVD, T2D, LD, and all-cause mortality) are mediated via alcohol’s effects on the gut microbiome and inflammatory mediators. And iii) assess associations between the functional potential of the gut microbiome and alcohol use.

## Materials and methods

### Sample selection

The national FINRISK surveys were a series of studies designed to measure and monitor common risk factors that affect population health in adult Finns [19]. These studies were conducted every five years between 1972 and 2012. The population of this current study was based on the FINRISK 2002 cohort (*n* = 8725). FINRISK adhered to the principles outlined in the Declaration of Helsinki [20], and received approval from the Ethical Committee on Epidemiology and Public Health of the Helsinki and Uusimaa Hospital District (decision number 87/2001). All participants gave informed written consent.

From the base sample, 7218 individuals had microbiome data available. Exclusion criteria were pregnancy (*n* = 40) and recent usage of antibiotics (*n* = 1307), which both greatly perturb the composition and function of the gut microbiome [21, 22]. The antibiotics included in the exclusions were antibacterials (ATC: J01), antimycotics (J02), antimycobacterials (J04), and antivirals for systemic use (J05), as classified by the WHO [23]. An individual was considered a user of antibiotics if they had a registered purchase within 6 months preceding the baseline investigation in the prescription medicine purchase register maintained by the Social Insurance Institution of Finland. Individuals were also excluded if they had missing phenotype or follow-up data (*n* = 1296). The final sample consisted of 4575 individuals after all exclusions (Supplemental Fig. 1).

### Follow-up period

Each permanent resident in Finland is given a personal identity number, which facilitates linking of study data with broader, nation-wide, public health registers. The registers ensure complete coverage of all significant health events over the course of an individual’s lifetime in Finland. In our study, the participants were followed through 31st of December 2019 (> 17 years from the baseline investigation). Only those participants who moved permanently abroad prior to experiencing health events or 31st of December 2019 were lost to follow-up. The reliability of public health registers in Finland has been verified [24, 25]. Health events pulled from these registers included purchases of prescribed medications and incidence information for CVD, T2D, LD, and all-cause mortality.

### Covariates and mediators

Covariates used in this study included age, sex, smoking status, dietary quality, body mass index (BMI), and usage of prescription medication known to cause shifts in gut microbiome composition/function. These data were gathered using a pre-sent questionnaire, at the baseline investigation, or via registers.

In the questionnaire, participants self-reported their smoking habits, and long-term diet information by filling out a food propensity questionnaire (FPQ). The questionnaire was checked by trained nurses at the baseline investigation. A participant was classified as a smoker if he/she reported daily smoking within six months prior to baseline investigation. Diet quality was quantified using a Healthy Food Choices (HFC) -score based on answers in the FPQ [26]. HFC assesses how well one’s diet conforms to a recommended diet, where a higher score indicates greater adherence. The score is a sum of the number of times per month an individual consumes dietary components that are viewed to be part of a recommended diet. The constituting components of the score are vegetables (incl. beans and lentils), fiber-rich breads, fruits, berries, fresh non-sweetened berry and fruit juices, fish, poultry, low-fat cheeses, salad dressings and oils, and nuts and seeds.

Anthropometric variables were measured at the baseline investigation. Height and weight were measured using established standards with light clothing and without shoes. BMI was calculated as weight (kg) divided by height (m) squared.

A participant was classified as a medication user if he/she had three consecutive purchases of any drug of interest in the drug-purchase register, the most recent of which had to be within 4 months prior to baseline investigation (not including antibiotics). The drugs of interest were metformin (ATC: A10BA02), drugs for constipation (A06A), psycholeptics (N05) and psychoanaleptics (N06) [23, 27, 28].

The variables for assessed potential mediators were sourced from blood samples drawn at the baseline investigation by trained nurses. These mediators included the plasma values for inflammatory markers C-reactive protein (CRP), interleukin-6 (IL-6), and tumor necrosis factor alpha (TNF-α).

### Data on alcohol use

Dietary information was gathered using the self-administered questionnaire, which also included questions on the use of various beverage types, including alcoholic ones. The query for alcohol intake asked for the number of consumed alcohol portions by alcohol types (12 g 100% alcohol/portion) per week over the past 12 months, which was converted into g 100% alcohol/wk. Correlation between alcohol use and smoking was *ρ* = 0.28 (Spearman’s rank correlation), and correlation with the HFC score was *ρ* = -0.22.

In analyses where data needed to be in categorical format, we first binned the data and then grouped the bins into one of three groups (low-, medium-, or high-risk use). We used the established national risk classification limits for alcohol listed in the Current Care Guidelines for upper bound bin limits and grouping [29–32]. Bin limits for the low-risk group were set at a maximum of 7 portions (84 g of 100% alcohol) per week for women and max. 14 portions (168 g) for men. Medium-risk group was set at max. 11 portions (132 g) per week for women and max. 22 (264 g) for men. The high-risk group was set at > 11 portions per week (> 132 g) for women and > 22 (> 264 g) for men.

### Fecal samples

Fecal samples were collected using at-home collection kits, that were returned with prepaid postal parcels via mail in typical Finnish winter conditions between Monday and Thursday. The samples were collected into 50 ml Falcon tubes without a stabilizing solution and were frozen immediately upon reception at -20 °C. Samples remained unthawed until sequencing at the University of California, San Diego.

A more detailed description of DNA-extraction, library preparation and sequencing of these samples has been reported [16]. In short, the samples were sequenced by a shallow-coverage shotgun metagenomics protocol, after which they were cleaned of human DNA reads and classified taxonomically by mapping the remaining reads against bacterial, archaeal, fungal, and viral genomic sequences. Final average read count was 900,000 reads per sample. Taxonomic annotation of the sequences was performed using Greengenes2 [33]. Functional annotation of each sample was performed with KEGG-orthology (KO) group relative abundances extracted using SHallow shOtGUN profiler (SHOGUN) *v1.0.5* (Knights Lab, University of Minnesota, Minneapolis, MN, USA) from strain-level outputs [34].

For analyses, we quantified associations of bacterial alpha- and beta-diversities, as well as divergences in individual bacterial species’ relative abundances. Alpha-diversity was quantified using Shannon index, which assesses both richness and evenness of the microbiome within a given sample [35]. Beta diversity, i.e. compositional similarity between samples, was quantified using weighted UniFrac distance [36, 37]. Weighted UniFrac distance is an extension of the UniFrac metric that accounts for both the phylogenetic relationships and the relative abundances of taxa. By including abundance information, it emphasizes differences in both the presence and prevalence of bacterial taxa. To analyze beta diversity with traditional regression methods, we divided the weighted UniFrac distances into principal coordinates (PCo), derived using principal coordinates analysis, which are a set of values that represent samples in a reduced-dimensional space based on their pairwise distances [38].

Alpha- and beta diversities were calculated from unfiltered species-level data. In species-specific analyses we filtered out rare species that had a prevalence under 1% and relative abundance under 0.01%. Species’ relative abundances were centered log-ratio-transformed (CLR) to account for their compositional nature [39]. KO-group data were standardized with a log10 transformation prior to analysis, and only statistically significant associations were selected for results curation.

### Statistical methods

Statistical work was performed in four steps using R version *4.3.2* [40], utilizing the package *mia* (version *1.2.7*) for microbiome data curation [41]. First, we checked for cross-sectional associations between alcohol use and the gut microbiome using multiple linear regression for alpha diversity, and PERMANOVA [42] and principal coordinates analysis [38] for beta diversity. For differential abundance analyses we utilized a linear regression framework with the ANCOM-BC2 package (version *3.18*), which corrects for unequal sampling fractions and differential sequencing efficiencies [43, 44]. Second, we ran prospective analyses to see whether alcohol use was associated with incident health outcomes using Cox proportional hazards regression. Third, we checked for potential mediation using Haye’s PROCESS-macro version *4.3.0* in R between alcohol use and health outcome associations via microbiome features alone or in series with inflammatory markers, using binary logistic regression to assess final associations with the health outcome of interest [45]. This was done for those alcohol-health outcome -associations that consistently gave a significant result in prospective models. Finally, we checked for functional associations by using multiple linear regression between each KO-group and alcohol use. Curation of the results was done using KEGG mapping tools provided by Kyoto Encyclopedia of Genes and Genomes on their website [46].

The validity of multiple linear regression models was checked by inspecting the distribution of their residuals. PERMANOVA was run with the number of permutation iterations set to 9 999. PERMANOVA runs were checked for homogeneity of variances with a dispersion test. Significant Cox models were checked for major violations to their assumptions of proportional hazards with a Schoenfeld test. Serial mediation analysis was performed with 10 000 bootstrap samples. To check which species contributed the most to beta diversity PCo values of interest, we calculated their correlation using Kendall’s tau and extracted the top species contributing to the respective PCo. We checked associations with the first 3 PCo’s in all applicable analyses.

All reported analyses were adjusted both demographically (Model 1; baseline age and sex) and with all covariates present in a fully adjusted configuration (Model 2; baseline age, sex, diet quality, smoking, medication, and BMI). Thus, model 2 is indicative of associations agnostic to influences of major gut microbiome perturbing factors. The effects of each covariate were assessed in intermediate models in a stepwise manner, but their results are not reported. Alcohol data and mediator variables were cubic-root-transformed to account for their skewed distributions. Continuous variables were scaled by Z-transformation prior to final analysis including data for covariates, mediators, alcohol use and CLR-abundances of bacterial species.

Significance threshold was set at a false-discovery-rate (FDR) -corrected q-value of < 0.05 for differential abundance analyses and functional analyses due to the large number of tests performed. The q-values were calculated using Benjamini-Hochberg FDR procedure [47]. A p-value of < 0.05 was used as a threshold for descriptive statistics, alpha diversity analyses, and beta diversity analyses. For prospective and mediation analyses a result was considered to be statistically significant if their respective 95% confidence interval excluded the value 1.

## Results

### Descriptive statistics

Women accounted for 52.5% of the sample. On average, men were older than women (49.3 vs. 47.5 years, Table 1) and slightly more overweight (BMI 27.2 vs. 26.5). Men also had an unhealthier diet (HFC 176.8 vs. 217.7), smoked more (28.7% vs. 20%), and used substantially more alcohol (124.8 vs. 47.7 g/wk.). Incidence of chronic diseases and all-cause mortality were also more common among men. Women were more commonly medication users (8.9% vs. 7.0%). Only variables where no differences were detected were incidence of LD and all three of the chosen inflammatory markers. The combined gut microbiome data of the whole cohort sample consisted of a total of 6324 species across 3038 genera and 992 families.The mean number of species per fecal sample was 1032.

**Table 1:** Descriptive statistics of the sample

Variable	Combined	Men	Women	*P*-value
n	4575 (100%)	2173 (47.5%)	2402 (52.5%)	
Age, years	48.4 ± 12.7	49.3 ± 12.7	47.5 ± 12.6	7.91 × 10 − 7
BMI, kg/m2	26.8 ± 4.6	27.2 ± 4.1	26.5 ± 5	2.86 × 10 − 8
HFC	198.3 ± 88	176.8 ± 80.5	217.7 ± 90	9.95 × 10–58
Smoking, n	1105 (24.2%)	624 (28.7%)	481 (20.0%)	6.95 × 10–12
Medication, n	365 (8.0%)	152 (7.0%)	213 (8.9%)	0.02
Alcohol, g/wk.	84.7 ± 121	124.8 ± 143.2	47.7 ± 80.1	5.41 × 10–99
CVD, n	510 (11.1%)	328 (15.1%)	182 (7.6%)	7.15 × 10–16
T2D, n	526 (11.5%)	287 (13.2%)	239 (10.0%)	5.62 × 10 − 4
Liver disease, n	87 (1.9%)	41 (1.9%)	46 (1.9%)	0.94
Death, n	552 (12.1%)	378 (17.4%)	174 (7.2%)	6.51 × 10–26
CRP, mg/l	2.3 ± 4.4	2.3 ± 4.5	2.4 ± 4.2	0.36
IL-6, ng/l	18.7 ± 147.5	19.5 ± 188.1	17.9 ± 88.5	0.83
TNF-α, ng/l	72.5 ± 673.8	93.3 ± 871.2	51.4 ± 380.8	0.23

Descriptive statistics for the study sample with all participants combined and stratified by sex. Values for continuous variables are means ± SD and for categorical variables the number of observations and their proportion in their respective population in parentheses. Groups were compared using Welch’s t-test for continuous variables and for categorical variables using Chi-squared test

### Alpha diversity

Mean ± SD values of alpha diversity in the different alcohol consumption groups were 4.11 ± 0.42 in the low-risk group, 4.07 ± 0.43 in the medium-risk group, and 4.04 ± 0.41 in the high-risk group. Alpha diversity was significantly lower in the high group when compared to the low group (*p* = 9.56 × 10^− 4^). Differences between other groups were non-significant (*p* > 0.05).

When adjusted for confounding factors in linear regression models, alcohol use had a negative association with alpha diversity (Coefficient: -0.015, SE: 0.007, *p* = 0.03) in model 1. This association did not survive the full adjustments of model 2 (Coefficient: -0.007, SE: 0.007, *p* = 0.33).

### Beta diversity

In PERMANOVA analyses, using weighted UniFrac distances, assessing differences in gut microbiome composition between individuals, alcohol use displayed a small yet consistent association with gut microbiome composition regardless of model setup (model 1, R^2^: 3.04 × 10^− 3^, Pseudo-F: 13.63, *p* ≤ 1.00 × 10^− 4^; model2, R^2^: 2.12 × 10^− 3^, Pseudo-F: 9.59, *p* ≤ 1.00 × 10^− 4^).

**Fig. 1:**
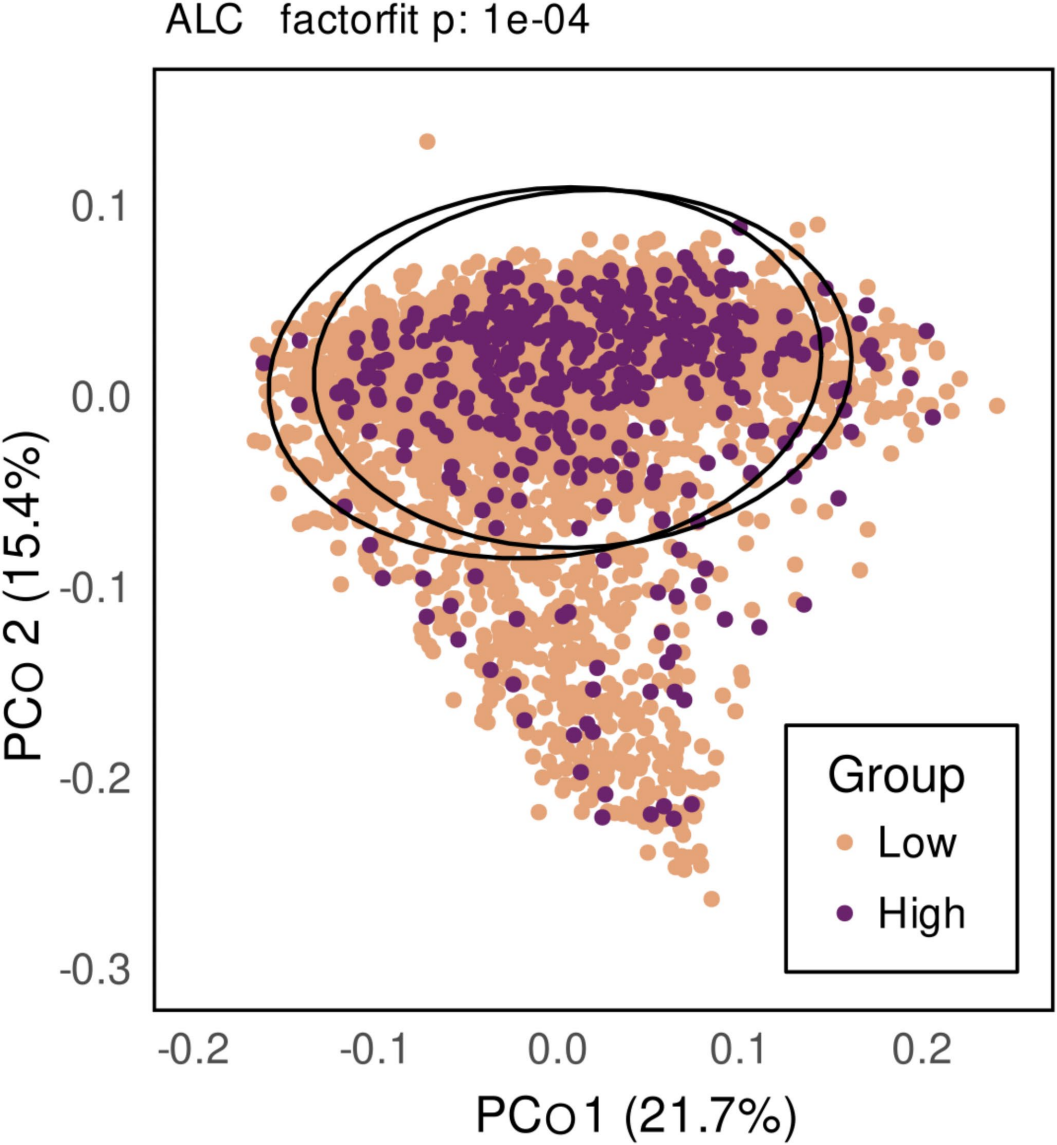
Ordination plot of the first two principal coordinates based on weighted UniFrac-distances of the samples stratified by low- and high-risk alcohol use. Individuals belonging to the low group are indicated with orange and individuals in the high group are indicated by purple. The P-value for a factorfit-test is displayed above the plot. Ellipsoids depict the spread of 95% of the samples within groups according to Student’s t-distribution. The amount of variance explained by each principal coordinate is marked on the axis labels

Ordination for PCo 1 (21.7% of variance explained) and PCo 2 (15.4%) displayed no clear clustering between low and high groups (Fig. 1). However, a factor fit test for the first three PCos (PCo 3: 8.2%) indicated significant divergence between the centroids of groups for alcohol use (r^2^:0.0046, *p* ≤ 1.00 × 10^− 4^), signaling a statistically significant difference in the gut microbiome compositions of low- and high-risk consumers of alcohol.

### Differential abundance analysis

Differential abundance analysis found significant associations for 344 species that displayed a consistent association regardless of model configuration (q < 0.05). Of these, 248 associations were positive and 96 negative. Notable taxa among the species that were negatively associated were multiple putatively beneficial species from the *Lactobacillus* (*n* = 6) and *Bifidobacterium* (*n* = 6) genera, and the species *Akkermansia muciniphila*. The positively associated group of bacterial taxa was dominated by members of the Gram-negative genera *Bacteroides* (*n* = 19) and *Prevotella* (*n* = 16). The top 10 species by the absolute value of their estimate in model 2 are displayed in Table 2. For a comprehensive listing of all significant species-level results in the fully adjusted model please see Supplemental Table 1.

**Table 2:** Differential abundance analysis results for top ten species

Species	Estimate	SE	Q-value
*Acinetobacter johnsonii*	-0.144	0.012	1.00 × 10^− 24^
*Bacteroides H cellulosilyticus*	0.125	0.021	1.53 × 10^− 8^
*Cryptobacteroides sp900316045*	-0.120	0.014	1.29 × 10^− 14^
*RUG705 sp900551455*	-0.113	0.018	2.92 × 10^− 9^
*Tidjanibacter inops A*	0.110	0.027	2.14 × 10^− 4^
*PeH17 sp000435055*	-0.108	0.022	9.48 × 10^− 6^
*Bifidobacterium bifidum*	-0.107	0.023	3.53 × 10^− 5^
*SFMI01 sp004556155*	-0.106	0.024	6.13 × 10^− 5^
*CAG-313 sp003539625*	-0.105	0.023	3.33 × 10^− 5^
*Parabacteroides B 862,066 distasonis*	0.105	0.018	5.37 × 10^− 8^

Model 2 results for top 10 species in descending order of absolute values of their estimates. Model 2 was adjusted for baseline age, sex, diet quality, smoking status, medication use, and BMI. Q-values were calculated from P-values with the Benjamini-Hochberg FDR-procedure

### Prospective analysis

When comparing incidence of health outcomes between the low and high groups, alcohol use displayed significant differences with LD (low: 1.5% vs. high: 4.4%, *p* = 1.72 × 10^− 5^) and all-cause mortality (low: 11.3% vs. high: 16.3%, *p* = 2.89 × 10^− 3^), after removing prevalent cases. CVD (low: 11.7% vs. high: 10.9%, *p* = 0.67) and T2D (low: 11.6% vs. high: 12.1%, *p* = 0.77) had no significant differences between groups.

**Fig. 2:**
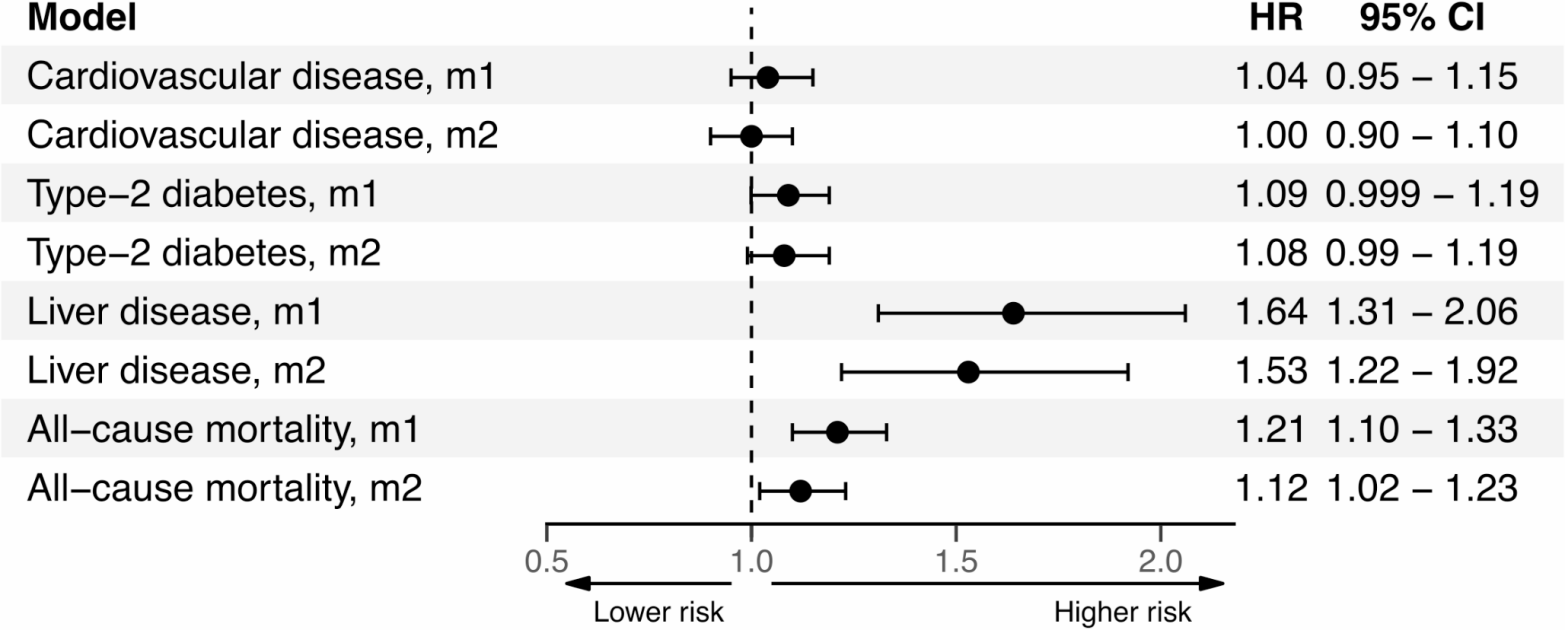
Forest plot for Cox-regression models assessing connections between alcohol use and incident health outcomes in two different model configurations. Model 1 (m1) included the covariates baseline age and sex. Model 2 (m2) included baseline age, sex, diet quality, smoking status, medication use, and BMI. Predictors were Z-transformed

**Fig. 3:**
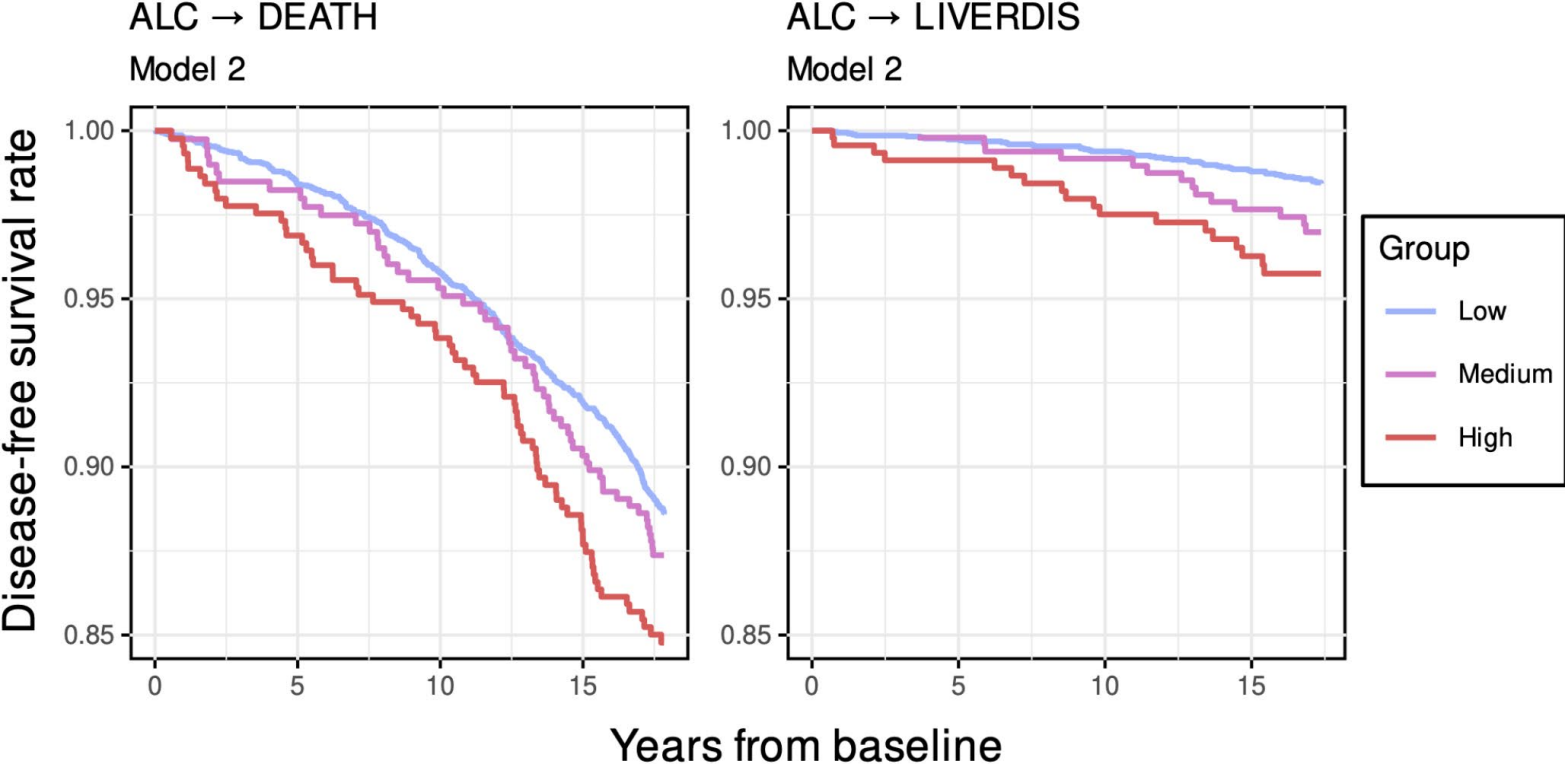
Adjusted survival curves for significant Cox-regression models assessing connections between alcohol use and all-cause mortality and incident liver disease stratified by usage group. On the y-axis is disease-free survival rate and on the x-axis is elapsed time from baseline investigation in years. The adjustment configuration for model 2 included baseline age, sex, diet quality, smoking status, medication use, and BMI

Cox regression corroborated these findings. Higher alcohol use associated with higher risk for LD (HR:1.53 [1.22—1.92], model 2) and all-cause mortality (HR:1.12 [1.02—1.23], model 2) in both model configurations (Figs. 2 and 3). Associations with CVD (HR:1.00 [0.90—1.10], model 2) and T2D (HR:1.08 [0.99—1.19], model 2) were non-significant in both model configurations (Fig. 2).

### Mediation analysis

**Fig. 4:**
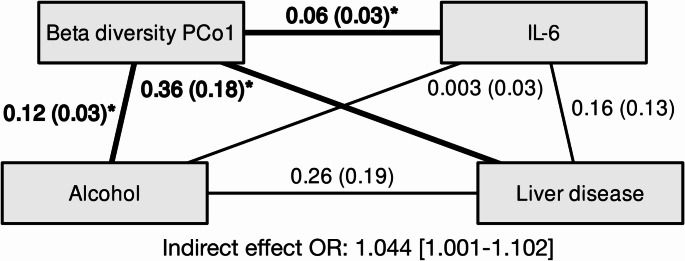
Bootstrapped standardized mean coefficients for associations between alcohol and liver disease mediated via beta diversity principal coordinate 1. The standard error for each respective coefficient is marked in parentheses. Statistically significant associations are in bold and marked with an asterisk. The proportion of the statistically significant indirect effect is marked below the figure as an odds ratio. Bootsrapping was set at 10 000 samples. Covariates (not displayed in the figure) included baseline age, sex, diet quality, smoking status, medication use, and BMI (model 2). Final associations with liver disease were assessed using binary logistic regression

Next, we assessed potential mediating connections involving microbial and inflammatory biomarkers with those alcohol-health outcome-associations that consistently displayed significant results across models in prospective analyses (i.e., LD and all-cause mortality). Across all possible model configurations (*n* = 48), we found one model that displayed consistent significant mediation between alcohol use and LD via microbiome beta diversity PCo 1 regardless of adjustment, with IL-6 included but not significantly involved in the mediation (Fig. 4). The mean bootstrapped OR for this indirect effect in model 1 was 1.05 [1.001—1.11] and in model 2 1.04 [1.001—1.10]. The total effects between alcohol use and LD in these models were 1.05 [1.01—1.11] and 1.05 [1.002—1.11], respectively. There were no significant direct effects.

Additionally, we observed three weak but significant serial mediations between alcohol use, microbiome, CRP and all-cause mortality via alpha diversity (OR: 1.0005 [1.00003—1.001]; direct effect: 1.26 [1.13—1.40]; total effect: ns.), beta diversity PCo 1 (OR: 1.001 [1.0004—1.002]; direct effect: 1.26 [1.13—1.40]; total effect: ns.), and beta diversity PCo 3 (OR: 1.0007 [1.0002—1.002]; direct effect: 1.25 [1.13—1.39]; total effect: 1.01 [1.004—1.02]). However, these mediations did not survive adjustment in model 2.

To find out which species contributed the most to PCo1, we extracted the top 10 species according to their absolute Kendall’s tau value. These species were the following: *Bacteroides_H uniformis* (τ: 0.450), *Scrofimicrobium canadense* (τ: 0.419), *Bacteroides_H sp007896885* (τ: 0.404), *Bacteroides_H fluxus* (τ: 0.387), *Cryptobacteroides pullicola* (τ: 0.378), *Bacteroides_H sp010501015* (τ: 0.372), *Bacteroides_H sp010500965* (τ: 0.369), *Bacteroides_G luti* (τ: 0.346), *Bacteroides_H ihuae* (τ: 0.341), and *Paramuribaculum sp900551515* (τ: 0.335). For a listing of the top 25 species contributing to PCo 1 please see Supplemental Table 2.

### Functional analysis

To gain more insight as to the potential mechanisms behind this mediating association, we performed a functional analysis. The analysis found 1643 significantly associated KO-groups (q ≤ 0.05), of which 431 had a positive and 1212 a negative association with alcohol consumption (Supplemental Table 3). Among the positively associated KO-groups, we identified 9 KEGG pathway modules that had all their constituting KO-groups significantly enriched. For the diminished KO-groups we identified 20 complete modules.

From the perspective of liver health, five modules were of particular interest, namely lipopolysaccharide (LPS) biosynthesis (M00063, enriched), phosphatidylcholine biosynthesis (M00091, diminished), pentose phosphate pathway (M00580, diminished), glutathione biosynthesis (M00118, diminished), and cysteine biosynthesis (M00338, diminished).

## Discussion

Our study found that alcohol displays associations with lower intraindividual microbial diversity, shifts in interindividual compositional makeup, and differences in the functional potential of the human gut microbiome. We also found an association between alcohol consumption and liver disease that has a mediating association via the gut microbiome. This mediation is possibly driven by a community-level shift towards a proinflammatory profile that is characterized by an increase in Gram-negative bacteria, a decrease in putatively beneficial species, and increased permeability of bacterial endotoxins through the intestinal barrier.

Pathological effects of alcohol on the human body are well known and risk profiles with various diseases have been thoroughly studied. Alcohol has been shown to have a positive curvilinear relationship with all-cause mortality, with a minimum mortality risk starting at a < 100 g per week threshold [3]. For cardiovascular diseases, alcohol consumption associates roughly linearly, and with no clear minimum risk threshold, with a higher risk for stroke, heart failure, coronary disease (excluding myocardial infarction), fatal hypertensive disease, and fatal aortic aneurysm, but with a lower risk for myocardial infarction [3]. As for type-2 diabetes, the relationship between temporal risk and alcohol consumption seems to follow a J-shaped association, with a slight decrease in risk observed at a consumption rate of approximately 10–14 g per day and an increase in risk observed above a 63 g per day consumption threshold [4]. However, this observed risk reduction for moderate consumers of alcohol might be specific only to women and non-Asian individuals. Finally, the association between alcohol consumption and liver diseases is well documented. Approximately 8–20% of heavy drinkers develop liver cirrhosis [5]. The risk profile for liver cirrhosis follows an exponential curve, the risk of which is more substantial for women than men, starting with one drink per day [9].

In the context of the gut microbiome the availability of epidemiological information is still limited, although some research is available from animal models and humans in smaller samples. Our findings that alcohol associates with proinflammatory and potentially health-detrimental compositional and functional shifts in the human gut microbiome fit current literature well. For instance, alcohol has been shown to associate with higher permeability of the intestinal wall and systemic inflammation, which is congruent with our findings [11]. Lower levels of *Akkermansia muciniphila*, which we also observed, following increased intake of alcohol, has been linked with dysregulated short-chain fatty acid (SCFA) production, impaired intestinal permeability, induction of chronic inflammation, and increased cytokine production [48]. Alcohol-associated liver disease and alcohol dependence have also been linked with reductions in *Lactobacillus* and *Bifidobacterium* species levels, respectively [49, 50]. Perhaps the clearest compositional change that has been observed in the gut microbiota following high alcohol intake and in individuals with moderate hepatitis is that of a community-level shift towards Gram-negative bacteria, along with a marked decrease in fecal concentrations of SCFAs and the relative abundances of their producers [51, 52]. Given that SCFAs facilitate the function of tight junctions between enterocytes, it is expected that a decrease in their producers is reflected in a deterioration of this function and an increase in inflammation [53, 54].

Our functional results highlight multiple biochemical functions that support the hypothesis that alcohol consumption associates with a proinflammatory gut microbiome profile and possibly also higher gut permeability, namely the enrichment of the LPS biosynthesis pathway and a diminished pathway for the biosynthesis of phosphatidylcholine, a key component in maintaining gut mucosal barrier integrity [55]. However, bacterial biosynthesis of phosphatidylcholine is relatively rare and there is scant evidence of the human gut microbiome producing de nuovo phosphatidylcholine in meaningful amounts. LPS, on the other hand, is a component of the outer membrane of Gram-negative bacteria, which constituted a large proportion of the positive species shift also in our study. When these bacteria proliferate, or when the integrity of the gut barrier is compromised, LPS and other endotoxic metabolites of microbial origin can translocate into the bloodstream and contribute to systemic inflammation, which is a known contributing factor to the pathogenesis of liver diseases such as alcoholic liver disease, hepatitis, and cirrhosis [11, 56, 57].

In addition, our study identified several negatively associated microbial pathways that were diminished with higher alcohol intake, and that are also of note in the context of liver health. Firstly, the pentose phosphate pathway is crucial for generating NADPH, which is an essential component in intracellular antioxidative processes [58]. Additionally, the pathways for the synthesis of cysteine and glutathione, both of which are also part of the intracellular antioxidative system, were also diminished [59]. A decline in the relative abundance of these pathways could indicate an impairment in the microbiome’s capability, especially that of Gram-positive bacteria, to withstand the oxidative stress caused by alcohol, which might contribute to dysbiosis of the gut microbiome and explain the observed associations with lower microbial diversity and community level shifts in composition.

### Strengths and limitations

Our study is the first one assessing epidemiological associations between alcohol, the human gut microbiome, and adverse health outcomes. Its prime strength is one of the largest available sample sizes of shallow-shotgun sequenced metagenomic gut microbiome cohorts available at this time, where a majority of other comparable studies have utilized 16 S rRNA sequencing. Our cohort is also well phenotyped, has a very long, > 17 years follow-up time, and is population-based, which make inferences for population health much more credible.

However, generalizing outside the sphere of the Nordic area should be done with caution due to differences in prevailing gut microbiome profiles and potential differences in the prevalence of liver diseases across different geographical areas [60]. Consequently, our results need to be verified in other cohorts. Alcohol use was self-reported, which might influence results, due to underreporting. Usage patterns and portion sizes for alcohol in Finland have also shifted since 2002, which to a degree complicates interpretation of our results. Finally, it is important to note that our analyses did not include adjustments for fecal transit time or physical activity, which both affect gut microbiome composition, and should be considered in future studies.

## Conclusions

We confirm in an epidemiological setting the findings of smaller scale studies that alcohol use is associated with a dysbiotic and proinflammatory gut microbiome profile, which is involved in the mediation of alcohol’s effect on liver diseases. This mediation is possibly facilitated by a decrease in relative abundances of putatively beneficial bacteria, an increase in endotoxemic bacteria, and systemic inflammation induced by increased gut permeability.

## Electronic supplementary material

Below is the link to the electronic supplementary material.

Supplementary Material 1

Supplementary Figure 1

## Data Availability

The dataset used is available upon request through the Findata permit procedure. https://www.findata.fi/en/. Analysis code is available at 10.5281/zenodo.10959582.
