# Supplementary material for: Associations of alcohol with the human gut microbiome and prospective health outcomes in the FINRISK 2002 cohort
Source: Eur J Nutr. 2025 Apr 11;64(4):153. doi: 10.1007/s00394-025-03668-z (PMC11991935; doi:10.1007/s00394-025-03668-z)
Supplement: Supplementary file 2 — Supplementary Figure 1 [file 394_2025_3668_MOESM2_ESM.pdf]

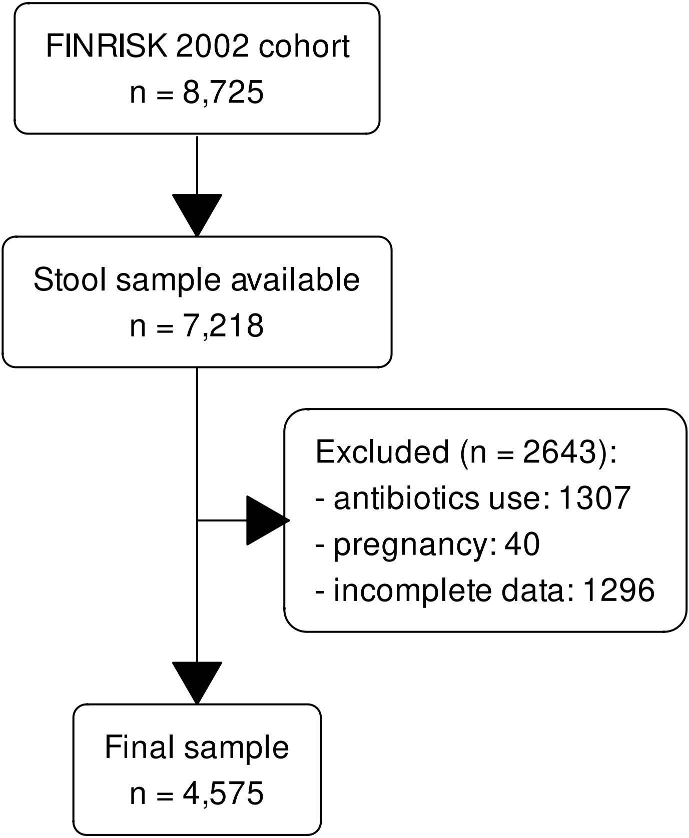
FINRISK 2002 cohort
n = 8,725
Stool sample available
n = 7,218
Excluded (n = 2643):
- antibiotics use: 1307
- pregnancy: 40
- incomplete data: 1296
Final sample
n = 4,575
